# Beyond the Surface: Mesophotic Reefs as Potential Refuges for Shallow Fish Assemblages

**DOI:** 10.1002/ece3.70619

**Published:** 2024-11-28

**Authors:** Manuel Francisco Velasco‐Lozano, Georgina Ramírez‐Ortiz, Luis Eduardo Calderon‐Aguilera, Benjamín Alonso Martínez‐Garza, Omar Valencia‐Méndez

**Affiliations:** ^1^ Departamento de Ecología Marina, Centro de Investigación Científica y de Educación Superior de Ensenada, Laboratorio de Esclerocronología Ecología y Pesquerías de la Zona Costera Ensenada Mexico; ^2^ Unidad Académica Mazatlán, Instituto de Ciencias del Mar y Limnología Laboratorio de Ecología funcional & conservación marina, Universidad Nacional Autónoma de Mexico Mazatlán Mexico; ^3^ División de Ciencias de la Salud, Biológicas y Ambientales Universidad Abierta y a Distancia de Mexico Mexico City Mexico

**Keywords:** deep reef refugia hypothesis, fish assemblages, marine protected areas, mesophotic zone, partial refugia, taxonomic and functional diversity

## Abstract

The deep reef refugia hypothesis suggests that the effects of disturbance decrease as depth increases; thus, reefs in the mesophotic zone potentially serve as refuges for communities in shallower zones. This study challenged this hypothesis by evaluating fish diversity in shallow and mesophotic reefs in a marine protected area in the Gulf of California. During 2021–2022, we conducted 189 5‐min video transects using remotely operated vehicles to document species richness and abundance. We evaluated six biological traits for each species (length, mobility, position, gregariousness, diet, and activity period) to estimate four functional indices (number of entities, richness, originality, and divergence), one phylogenetic index (Δ*), and Hill's numbers for taxonomic and functional indices. Benthic organisms were analyzed to explore relationships with ichthyofauna, while monthly water turbidity satellite data products were transformed into a light attenuation coefficient to identify the mesophotic zone (area between 10% and 0.1% of the incident light at the surface). At the study site, the mesophotic zone was identified to extend to 21 m under optimal conditions, which is shallower than what is typically observed in oligotrophic regions. Generalized linear models revealed significant variations in reef fish composition across spatial (site and zone) and temporal (season and year) dimensions. Additionally, generalized linear mixed models of functional richness and taxonomic Hill's numbers exhibited significantly higher values in the shallow zone. However, functional and phylogenetic indices showed similarities in fish assemblages. Despite differences in fish taxonomic diversity among zones that could be related to less environmental variation and resource availability in deep strata, mesophotic reef fish assemblages presented similar functions. Functions were maintained in mesophotic reefs, which suggests that the two zones are connected and that mesophotic reefs have the potential to act as partial refugia in the face of current and near‐future climate change‐related disturbances that could affect shallow zones.

## Abstract 2

Mesophotic reefs at PNZMAES could be considered partial refugia since they present different species but host shared species with similar functions as shallow reefs.

## 1 Introduction

Refuges are habitats that provide spatial and temporal protection from disturbances that occur in relatively short timescales (minutes to decades); therefore, they are considered buffers against potential local‐scale changes in resource availability and biodiversity (Keppel et al. 2012; White and Pickett 1985). Deep coral reefs have been extensively studied among marine habitats to assess their potential as refuges, leading to the proposal of the deep reef refugia hypothesis (DRRH). This hypothesis states that natural and anthropogenic impacts decrease as depth increases, making deep reefs potential refuges for shallow communities due to the stability of their environmental conditions and connectivity between their populations (Bongaerts et al. 2010; Bongaerts and Smith 2019; Glynn 1996).

In addition to coral reefs, fish‐associated fauna are one of the most studied groups in deep habitats (Ajemian et al. 2015; Pyle et al. 2019). Recently, Loiseau et al. (2022) proposed five different categories to describe the refuge potential of deep reefs based on fish assemblages: (1) not refugia (the taxonomic and functional diversity of fish in shallow and deep reefs are completely different), (2) partial refugia (deep reefs are equally or less diverse than shallow reefs, but both contain exclusive species and traits), (3) functional refugia (only functional diversity is similar between shallow and deep reefs), (4) full refugia (shallow and deep reefs exhibit the same taxonomic and functional diversity), and (5) enriched refugia (deep reefs have the same diversity as shallow reefs and also contain exclusive species and traits). When analyzing a particular case in the Indian Ocean, the authors found that functional richness was similar between shallow and deep reefs, despite the significant decrease in fish species richness and biomass as depth increased. This result suggested that deep reefs (< 100 m) might act as functional refugia for shallow reefs.

Traditionally, deep reefs that are located between 30 and 150 m have been categorized as mesophotic reefs (Kahng et al. 2019), which are presumed to have the highest potential to function as refuges, as they receive between 10% and 0.1% of the incident light at the surface and offer similar habitats (e.g., the presence of coral colonies and rocky substrate) to those of shallow reefs (Laverick et al. 2020; Pyle and Copus 2019). However, this assumption remains debatable, as does the definition of the bathymetric range for the mesophotic zone, which can vary according to environmental factors, such as light penetration, water quality, temperature, and runoff, as well as biological factors, such as eutrophication and phytoplankton productivity (Kahng et al. 2019; Tamir et al. 2019; Zarubin, Lindemann, and Genin 2017). Thus, it is essential to determine the depth range for the mesophotic zone in regions with distinct environmental and biological factors to properly identify mesophotic ecosystems and evaluate their potential to act as refuges for shallow communities in the near future.

Characterizations of mesophotic habitats and their associated fauna have increased in number and expanded in scope, thanks to the use of remotely operated vehicles (ROVs), which are highly versatile, given that they allow for the joint use of cameras, environmental sensors, and sampling devices (Armstrong, Pizarro, and Roman 2019; Locker et al. 2010). Nonetheless, the plethora of ROV studies has been limited to oligotrophic regions, such as the Caribbean Sea and the Gulf of Mexico (Laverick et al. 2018; Turner et al. 2017) due to their low primary productivity (mean: 249 mg C m^−2^ d^−1^) and, consequently, high water clarity and low drift (e.g., currents and waves). These characteristics have facilitated navigation and operation, allowing ROVs to explore coral reef ecosystems at great depths (Garrison 2009; Karleskint, Turner, and Small 2010).

In contrast, eutrophic regions, such as the Eastern Tropical Pacific (mean primary productivity: 376 mg C m^−2^ d^−1^), exhibit highly variable wind, swell, and current conditions throughout the year (Fiedler and Lavín 2017; Pennington et al. 2006). For this reason, these regions have remained among the least explored via ROVs, given the challenges posed for their operation and navigation. Nonetheless, a few studies have been conducted in the mesophotic habitats of the Gulf of California and Revillagigedo Archipelago that have analyzed the relationships between environmental factors and biological communities.

Hollarsmith et al. (2020) evaluated the effect of environmental factors on fish species composition between 12 m and 94 m. These authors stated that habitat type, temperature, and depth affected species composition and reported that shallow (< 30 m) and mesophotic zones (> 30 m) exhibited 60% resemblance. Velasco‐Lozano et al. (2020) compared fish species and functional richness between the mesophotic habitats (rocky reefs and sandy bottoms) of continental and oceanic islands. The authors found significant differences between habitats for all the ecological indicators, although functional richness in oceanic islands varied due to the presence of transpacific species and concluded that fish diversity at mesophotic depths is influenced more by the structural complexity of the sea floor than by the notable anthropogenic disturbances that could affect the continental islands due to their closeness to urban areas. Finally, Silva‐Montoya, Ramírez‐Ortiz, and Calderon‐Aguilera (2024) compared the functional and trophic structure of fish communities in shallow and mesophotic reefs and found that species richness and density decreased with depth, which could be associated with lower temperature and light conditions that limits primary productivity (the principal food resource for herbivores). Nonetheless, the functional structure of the fish communities was similar and biomass was greater between depth zones; thus, mesophotic habitats could act as refuges, especially for commercial species.

Based on the above, the present study aimed to determine the depth range of the mesophotic zone at *Parque Nacional Zona Marina del Archipiélago de Espíritu Santo* (PNZMAES), a marine protected area in the Gulf of California, and to evaluate its mesophotic habitats as potential refuges by comparing the taxonomic, phylogenetic, and functional diversity of reef fish assemblages among depth strata and its relationship with benthic species. Since PNZMAES is located within an area of high primary productivity, we hypothesized that the mesophotic zone would be shallower (< 30 m) than in oligotrophic regions where light penetrates more deeply within the water column. Due to high spatial proximity and connectivity among zones, we also hypothesized that the taxonomic, phylogenetic, and functional diversity of the fish communities would be similar among depth zones. Thus, the mesophotic habitats in this region could act as full refugia, especially considering the current disturbances that threaten shallow zones.

## 2 Methods

### 2.1 Study Area

**FIGURE 1:**
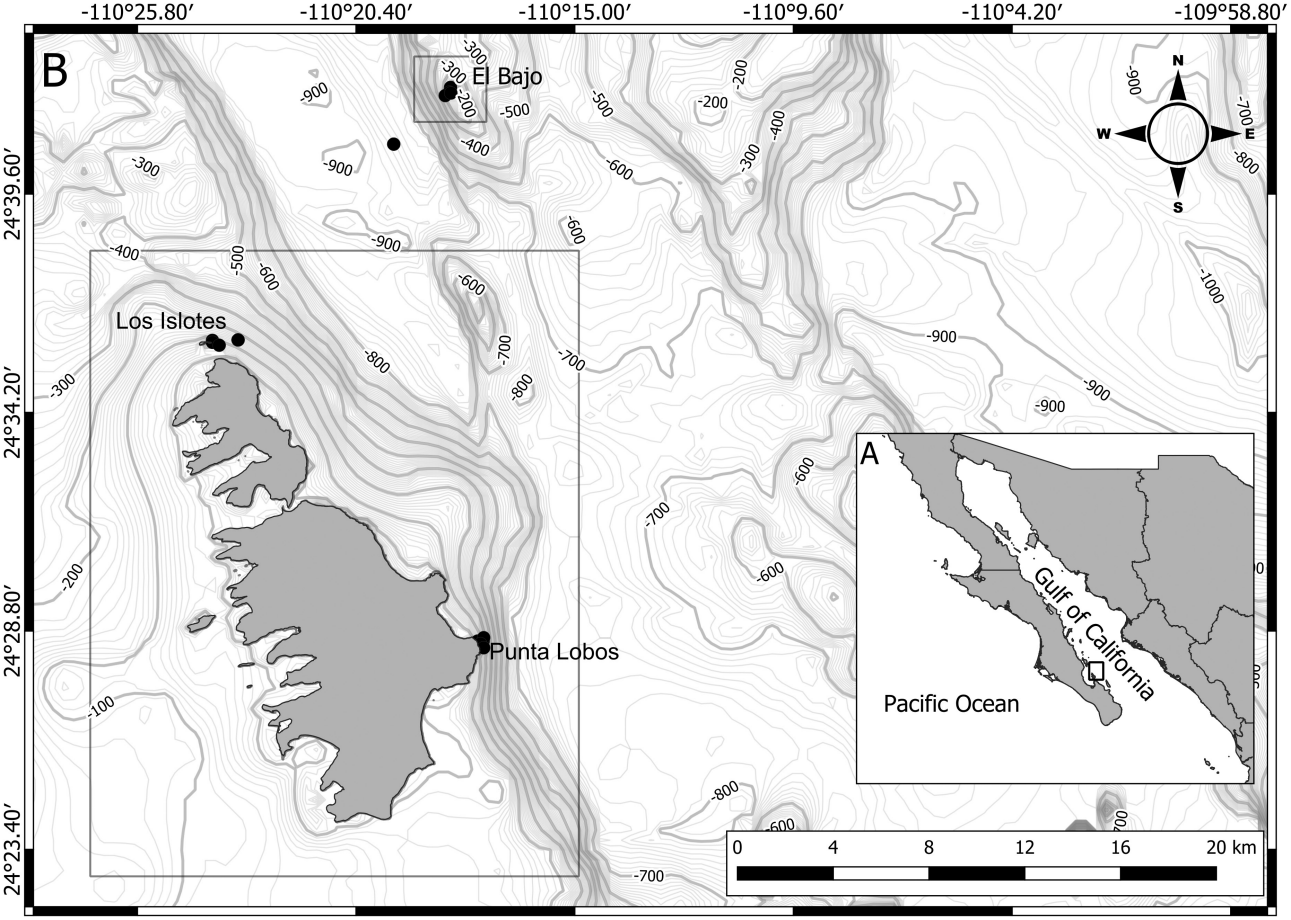
*Parque Nacional Zona Marina del Archipiélago de Espíritu Santo* (PNZMAES) depicting (A) the location of the marine protected area in the Gulf of California and (B) the protection polygon. The locations of the survey sites are shown with black dots.

PNZMAES is located in the southwestern Gulf of California (Figure 1; CONANP 2014). The North American monsoon influences this area of the gulf, which exhibits semi‐arid conditions, characterized by intense northerly winds during winter (8–12 m s^−1^) and weak southerly winds (5 m s^−1^), in addition to tropical storms that increase cloud cover, precipitation, and terrigenous discharge during summer (Monreal‐Gómez, Molina‐Cruz, and Salas‐de‐León 2001; Herrera‐Cervantes 2019).

The oceanographic conditions of the study area are driven by two surface water masses (tropical surface water [TSW] and Gulf of California water [GCW]) and a deep‐water mass (subtropical subsurface water [StSwW] between 150 and 500 m), the confluence of which makes the area a transitional zone of intense mixing (Lavín and Marinone 2003; Castro et al. 2006; Portela et al. 2016). The cold season is characterized by high nutrient concentrations, low sea surface temperature (~20°C), and water‐column stratification, whereas the warm season is characterized by low nutrient concentrations, high sea surface temperature (~30°C), and stratification with a mixed layer (Reyes‐Salinas et al. 2003; Obeso‐Nieblas et al. 2007; Villaseñor‐Casales 1979).

The protected polygon of PNZMAES comprises a total area of 486.54 km^2^ (6.66 km^2^ of no‐take zones and 479.88 km^2^ of buffer zones; CONANP 2014). Recently, PNZMAES was recertified in the Green List of Protected Areas of the International Union for Conservation of Nature (UNEP‐WCMC and IUCN 2024). This study was conducted in two no‐take zones (Punta Lobos and Los Islotes) and one buffer zone (El Bajo de Espíritu Santo [El Bajo]). The main habitat features of these sites are large boulder patches and walls, which provide ideal habitats for fish to take shelter and reproduce (Aburto‐Oropeza and Balart 2001; Richert et al. 2017). In particular, El Bajo stands out for harboring a high diversity of fishes due to the high primary productivity associated with upwelling near seamount peaks (~20 m; Amador‐Buenrostro et al. 2003; CONANP 2014; Trasviña‐Castro et al. 2003).

The sites in this study have been explored with ROVs since 2018. Therefore, this project marks the beginning of a monitoring program for mesophotic reefs and an extension of the shallow monitoring program of PNZMAES, which has been in operation since 2005 (CONANP 2014).

### 2.2 Bathymetric Range of the Mesophotic Zone

On the eastern coast of PNZMAES, we estimated the light attenuation coefficient (*K*_dPAR_) to determine the location of the mesophotic zone; *K*_dPAR_ indicates the decomposition rate of photosynthetically active radiation throughout the water column and is a measure of transparency. A level 4 water turbidity data (*K*_d490_) product was compiled from the Copernicus Marine Service using a monthly 4‐km spatial resolution. The values for each site were transformed into *K*_dPAR_ using the equation proposed by Morel et al. (2007) for Case I waters (< 0.2 m^−1^):

(1) $$K_{\text{dPAR}}=0.0864+0.0884\;K_{d490}-0.00137\;{\left[K_{d490}\right]}^{-1}$$

Based on these values, the bathymetric range of the mesophotic zone at 10%, 1%, and 0.1% of the incident light at the surface was estimated using the calculations proposed by Pérez‐Castro et al. (2022), where *K*_d1_ and *K*_d2_ are the minimum and maximum mean *K*_dPAR_ values per month, respectively:

(2) $$z_{10\%}=\frac{2.3}{K_{d2}}$$

(3) $$z_{1\%}=\frac{4.6}{\left(K_{d1}+K_{d2}\right)/2}$$

(4) $$z_{0.1\%}=\frac{6.9}{K_{d1}}$$

Optical depth at 10%, 1%, and 0.1% of the incident light at the surface were considered the upper limit, midpoint, and lower limit, respectively, of the mesophotic zone (Kirk 2011; Laverick et al. 2020; Pérez‐Castro et al. 2022). Data wrangling and processing were conducted with the ‘tidyverse,’ ‘raster,’ and ‘ncdf4’ packages in R (Hijmans et al. 2023; Pierce 2023; R Core Team 2023; Wickham et al. 2019).

### 2.3 Data Collection

Reef exploration and species surveys were conducted during June 2021 and April 2022 (cold season) and October 2021 and 2022 (warm season) using two ROVs. During 2021, the surveys were carried out with a VideoRay Pro 4 (VideoRay, Pottstown, PA, USA), which is capable of diving to 300 m and is equipped with depth and temperature sensors, compass, 3600‐lm LED lights, three thrusters, 150‐m tether cable, and a primary camera (VideoRay 2010). In 2022, surveys were conducted with a BlueRov2 (Blue Robotics, St. Torrence, CA, USA), which is capable of reaching 200 m and is equipped with a depth and temperature sensor, six thrusters, four 1500‐lm LED lights, 150‐m tether cable, and a primary camera (Blue Robotics 2023). A GoPro HERO 3 (GoPro, San Mateo, USA) seabed‐facing camera (medium‐angle setting) was installed on both ROVs.

Each dive lasted a total of 60 min in each zone (shallow and mesophotic) and site (Punta Lobos, Los Islotes, and El Bajo), regardless of environmental conditions. Afterward, the primary camera and GoPro footage were synchronized and trimmed into five‐minute sections, beginning when the ROV reached the sea floor. A period of 1 min between sections was included to ensure sample independence and avoid pseudoreplication issues (Hurlbert 1984). In all, 189 sampling units or video transects (2021 *n* = 94; 2022 *n* = 95) were obtained over both years. In each video transect, the percentage of sand (particles < 0.5 cm), gravel (0.5–15 cm), block (> 15 and < 100 cm), and rock (> 100 cm) were estimated with point‐intercept transects (PIT), with the occurrence of each substrate category recorded every 10 s (*n* = 30 per sampling unit). As a measure of vertical complexity, the normalized number of soft corals (*Octocorallia*) and black corals (*Antipathes galapagensis*), along with the percentage cover of hard coral (*Hexacorallia*), were also estimated by PIT.

Footage editing and visualization to register fish species were performed with CyberLink Power DVD v. 17 software (CyberLink, New Taipei City, Taiwan).

### 2.4 Data Analyses

In each video transect, all fishes within the visual field of the cameras were identified to the minimum possible taxonomic level using the Eastern Tropical Pacific fish guide; their valid scientific names were confirmed using Eschmeyer's Catalog of Fishes (Fricke, Eschmeyer, and van der Laan 2023; Robertson and Allen 2015). Fish records were not included when currents dragged the ROV or when organisms swam parallel to the vehicle, as these individuals in these records could have been registered previously. After eliminating these records, species richness (S) and abundance (N) per video transect were estimated. Fish records were plotted as a Venn diagram to visualize shared and exclusive species between bathymetric zones using the ‘tidyverse’ and ‘ggVennDiagram’ packages in R (Wickham et al. 2019; Gao et al. 2024).

Abundance data were $\sqrt[{4}]{x}$ transformed to down‐weight the more abundant species before generating a Bray–Curtis dissimilarity matrix. Afterward, a nonmetric multidimensional scaling (nMDS) analysis was performed with the ‘vegan’ package in R (Oksanen et al. 2001) to depict similarities in reef fish composition between zones (shallow and mesophotic), which were encompassed by ellipses that indicated the 95% confidence interval. The type of substrate (sand, gravel, rock, and block) and the normalized abundance of hard corals, soft corals, and black corals were incorporated as vectors in the nMDS plot to visualize patterns between fish diversity and habitat complexity in each zone. To improve the clarity of the visualization, the transects with no data and outliers were removed, and the vectors were scaled.

To assess the effect of zone, site (Punta Lobos, Los Islotes, and El Bajo), season (warm and cold), year (2021 and 2022), and the interaction between them, a model‐based analysis for multivariate abundance data was performed (Wang et al. 2012; Bosch et al. 2021). This method offers advantages over traditional distance‐based analyses (e.g., PERMANOVA) as it involves creating generalized linear models (GLMs) per species to make multivariate inferences that are unlikely to fail to meet the assumptions of correlation analysis (Wang et al. 2012). GLMs with negative binomial distributions and *p*‐values (999 iterations) were obtained via PIT‐trap resampling using the ‘tidyverse’ and ‘mvabunds’ packages in R (Wang et al. 2012; Wickham et al. 2019).

The following six traits were considered to be proxies of the ecological roles of each species based on previous regional studies (Olivier et al. 2018; Ramírez‐Ortiz et al. 2020) and the information in Fishbase (Froese and Pauly 2023): maximum length (ordinal: 0–7, 7–15, 15–30, 30–50, 50–80, and > 80 cm), mobility (ordinal: highly attached to the site, mobile with a small home range, mobile with an extensive home range, and highly mobile with an extensive home range), activity period (nominal: day or night), gregariousness (ordinal: solitary, in pairs, small schools, and large schools), position in the water column (ordinal: benthic, benthopelagic, and pelagic), and diet (nominal: herbivore–detritivore, invertivore of sessile organisms, invertivore of mobile organisms, planktivore, piscivore, and omnivore). By pooling the attributes together per species, an alphanumeric code was generated, which was defined as the functional entity (FE; Appendix S1). The FE indicates if one or more species share categories for a trait (Mouillot et al. 2013, 2014).

The attribute matrix was transformed into a quantitative similarity matrix using Gower's distance coefficient, which allows nominal and ordinal variables to be compared and gives them equal weight (Gower 1971). Only the video transects in which species richness was higher than the number of traits were considered in the analysis (*S* ≥ 6 species), as functional richness and divergence cannot be estimated with a lower number of species than traits (Villéger, Mason, and Mouillot 2008).

Distances between species were transformed into coordinates using a principal coordinate analysis (PCoA), and these values were used to build a functional space known as a convex hull, which was determined by the outermost vertexes or atypical traits (Cornwell, Schwilk, and Ackerly 2006; Mouchet et al. 2010; Villéger, Mason, and Mouillot 2008). Only four coordinate values to represent the functional space were selected based on the criteria of Mouillot et al. (2021), given that the percentage of explained variance was similar between four (75%) and five dimensions (83%; Magneville et al. 2023). Species richness (No. spp.), number of FEs (No. FE), and functional volume (Vol.) of the fish assemblages in shallow (≤ 21 m) and mesophotic (> 21 m) habitats were visualized in plots to identify differences between bathymetric zones.

Coordinate values and abundance per species were also used to calculate three functional indices per video transect: (a) functional richness (FRic), which indicates the distribution of FEs and the amount of functional space covered by the fish assemblage in each video transect in proportion to the total volume (Mouchet et al. 2010; Villéger, Mason, and Mouillot 2008); (b) functional originality (FOri), which represents the isolation of FEs within the functional space weighted by abundance and allows the functional redundancy of the assemblage to be quantified (Mouillot et al. 2013); and (c) functional divergence (FDiv), which describes the mean distance of each FE to the centroid of the functional space weighted by abundance (Mouillot et al. 2013; Villéger, Mason, and Mouillot 2008).

The Hill numbers were also calculated to measure taxonomic and functional diversity using the ‘mFD’ package in R (Magneville et al. 2023). This framework unifies diversity measures because they are equivalent to the transformations of other ecological indicators; it also summarizes diversity into a single unit and allows for direct comparisons between studies due to mathematical principles and properties (e.g., replication principle and doubling property; Chao, Chiu, and Jost 2014; Gotelli and Ellison 2013). Hill numbers are determined by the parameter *q*, which defines the sensitivity to the relative abundance of each species or FE: high *q* values suggest more evenness among abundances, whereas low *q* values indicate the dominance of some species or FEs (Chao et al. 2019; Gotelli and Ellison 2013). Taxonomic and functional diversity were expressed as richness (*q* = 0) and entropy (*q* = 1; number of typical species or FEs), respectively.

Following a phylogenetic approach, the taxonomic distinctness index (Δ*) was estimated for each video transect. This index assesses the relatedness among the species of an assemblage according to the Linnaean classification tree, with six hierarchical levels (genus, family, order, subclass, class, and superclass; Clarke and Warwick 1998). This measure allowed assemblages to be compared among zones by detecting the differences in species identity and abundance (Clarke and Warwick 1998). Additionally, a 95% confidence funnel was plotted to visualize if the value of Δ* per video transect was lower than expected, which would indicate high relatedness among the species present within the assemblage. This diversity measure was obtained with the ‘vegan’ package in R, whereas the funnel plot was obtained with the ‘tidyverse’ package in R (Oksanen et al. 2001; Wickham et al. 2019).

Taxonomic, functional, and phylogenetic indices were used as complementary indicators to generate a comprehensive description of biodiversity in terms of taxonomic identities, functional characteristics, and evolutionary relatedness of the species in shallow and mesophotic depths (Bosch et al. 2021). These diversity indicators were fitted in generalized linear mixed models (GLMMs) to determine spatial differences in fish assemblages between zones, following Poisson (species richness) and Gamma (rest of the indices) distributions. Diversity indices were considered response variables, and zone was considered a fixed factor. Year, season, and site were considered random factors to account for the natural spatiotemporal variability of the data (Crawley 2013; Beckerman, Childs, and Petchey 2017). The GLMMs were created with the ‘lme4’ package, and model assumptions were visually inspected with the ‘ggResidpanel’ package in R (Bates et al. 2015; Goode and Rey 2022). Significant differences (95% confidence interval) between zones were determined at *p* < 0.05 for each taxonomic and functional index. The refuge potential of deep reefs was based on the categories proposed by Loiseau et al. (2022): (1) not refugia (the taxonomic and functional diversity of fish in shallow and deep reefs are completely different), (2) partial refugia (deep reefs are equally or less diverse than shallow reefs, but both contain exclusive species and traits), (3) functional refugia (only functional diversity is similar between shallow and deep reefs), (4) full refugia (shallow and deep reefs exhibit the same taxonomic and functional diversity), and (5) enriched refugia (deep reefs have the same diversity as shallow reefs and also contain exclusive species and traits).

All data transformation, diversity index calculations, and plots were performed or generated with the ‘tidyverse,’ ‘mFD,’ ‘geometry,’ and ‘tripack’ packages in R (Magneville et al. 2023; Renka et al. 2020; Roussel et al. 2023; Wickham et al. 2019).

## 3 Results

During the 2021–2022 study period, similar average values at each site were observed for water transparency (*K*_d490_ > 0.07 m^−1^; *K*_dPAR_ > 0.13 m^−1^). For both coefficients, higher values were observed during the cold season (*K*_d490_ > 0.09 m^−1^; *K*_dPAR_ > 0.15 m^−1^) compared to those recorded during the warm season (*K*_d490_ > 0.04 m^−1^; *K*_dPAR_ > 0.09 m^−1^). As a result, optical depths at 10%, 1%, and 0.1% of the incident light at the surface varied throughout the year, with the mesophotic zone found between 11 m and 37 m during the cold season and between 21 m and 67 m during the warm season (Appendix S2). Based on these results, video transects above 21 m were considered shallow surveys, whereas those below 21 m were considered mesophotic surveys.

A total of 96 species were identified from 189 video transects in the shallow and mesophotic zones of PNZMAES during 2021–2022, belonging to 65 genera and 35 families of the classes Chondrichthyes (8 species) and Actinopterygii (88 species). Of the total, 52 species (54%) were shared between zones; 29 species were exclusive of the shallow zone, whereas 15 were only present in the mesophotic zone (Appendices S1 and S4). With regard to biological traits, the species observed in shallow reefs were grouped into 62 FE and their dominant traits were medium‐large size (> 15 cm), high mobility (within and between reefs), diurnal activity, solitary behavior, and benthic preference, as well as diets mainly composed of mobile invertebrates and fishes (e.g., *Mycteroperca rosacea* and *Balistes polylepis* ; Appendix S1). Likewise, 50 FEs were registered in mesophotic reefs, with similar dominant traits to those reported for shallow fish communities but with different representative species (e.g., *Hoplopagrus guentherii* and *Haemulon sexfasciatum* ). Large‐sized, highly mobile, and invertivore and piscivore species, such as *Cephalopholis colonus*, *Holacanthus passer* , and *Lutjanus argentiventris* , were recorded in both strata.

**FIGURE 2:**
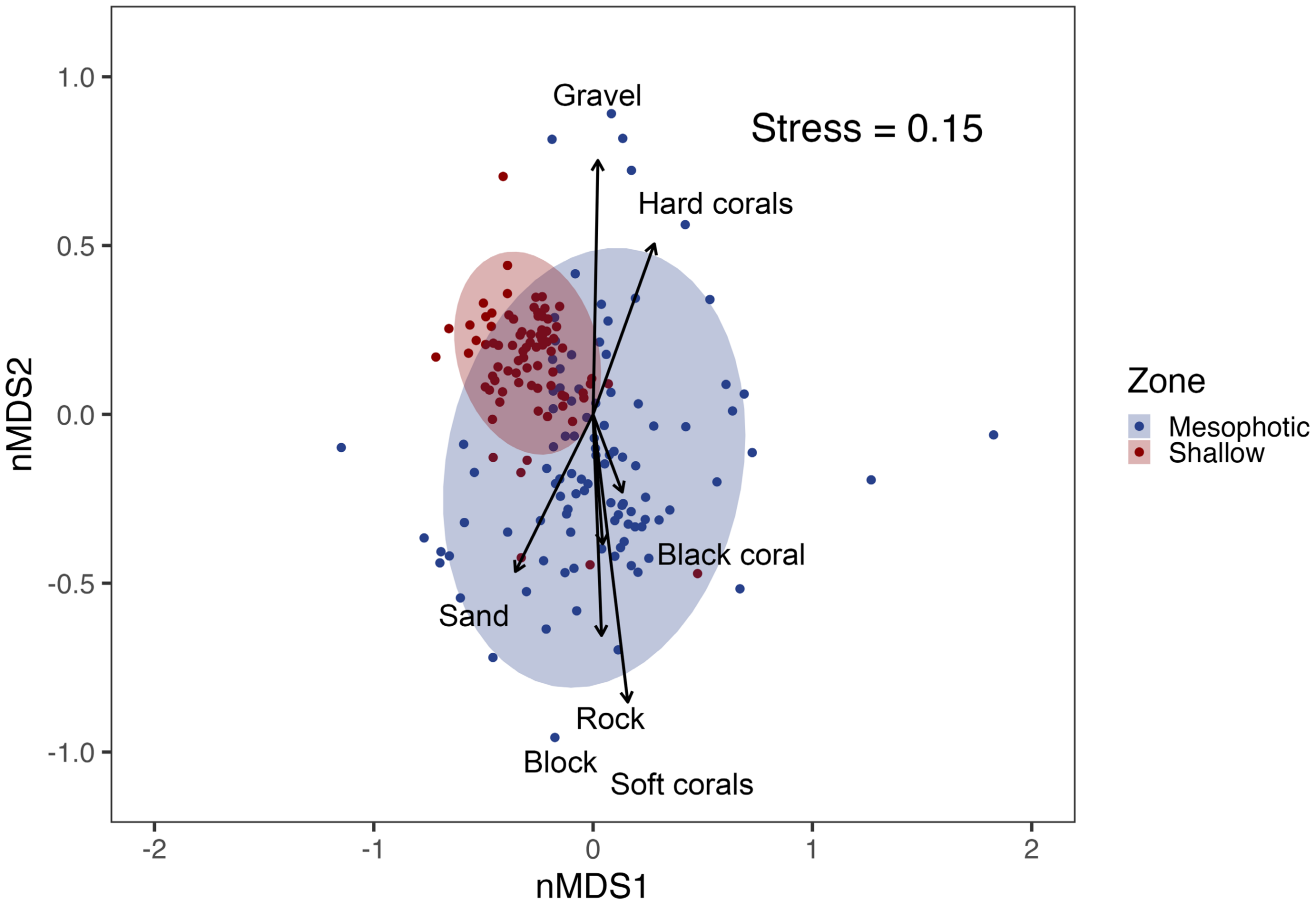
Nonmetric multidimensional scaling analysis (nMDS; Bray–Curtis coefficient) based on the presence and abundance (fourth‐root transformed) of fish species in each transect in shallow (red) and mesophotic (blue) reefs in *Parque Nacional Zona Marina del Archipiélago de Espíritu Santo* (PNZMAES) during 2021–2022. Ellipses indicate the 95% confidence intervals, while vectors illustrate the substrate (sand, gravel, block, and rock) and vertical complexity (soft corals, black corals, and hard corals) categories (none of them had a significant effect on fish diversity).

The nMDS analysis revealed a high resemblance in fish assemblages among depths, with no effects on the substrates or vertical complexity categories. Despite this, further investigation is necessary to determine the relationship between soft and black corals and mesophotic fish diversity as a positive, non‐significant relationship was detected between these two factors. In addition, shallow fish assemblages appeared to be more related to sandy and rocky substrates (Figure 2).

According to the GLM, significant differences in reef fish composition at PNZMAES could not be attributed only to depth; the factors of year, season, and site (and some of the interactions between them) also played a role (*p* < 0.03; Table 1). These results suggest that the composition of fish assemblages varies over space (site and zone) and time (season and year).

**TABLE 1:** Generalized linear models results accounting for differences in reef fish composition between zone, year, season, and site at Parque Nacional Zona Marina del Archipiélago de Espíritu Santo.

Factors	Residual degrees of freedom	Deviance	*p*
Zone	187	1164.40	0.001*
Year	186	301.90	0.001*
Season	185	333.90	0.001*
Site	183	1141.90	0.001*
Zone × Year	182	233.50	0.002*
Zone × Season	181	148.30	0.001*
Year × Season	180	163.40	0.001*
Zone × Site	178	314.60	0.001*
Year Site	167	294.90	0.003*
Season × Site	174	328.60	0.005*
Zone × Year × Season	173	73.60	0.009*
Zone × Year × Site	171	242.10	0.023*
Zone × Season × Site	169	37.70	0.003*
Year × Season × Site	167	0.00	0.571
Zone × Year × Season × Site	170	0.00	0.233

* Significant effect of the analyzed factors.

**FIGURE 3:**
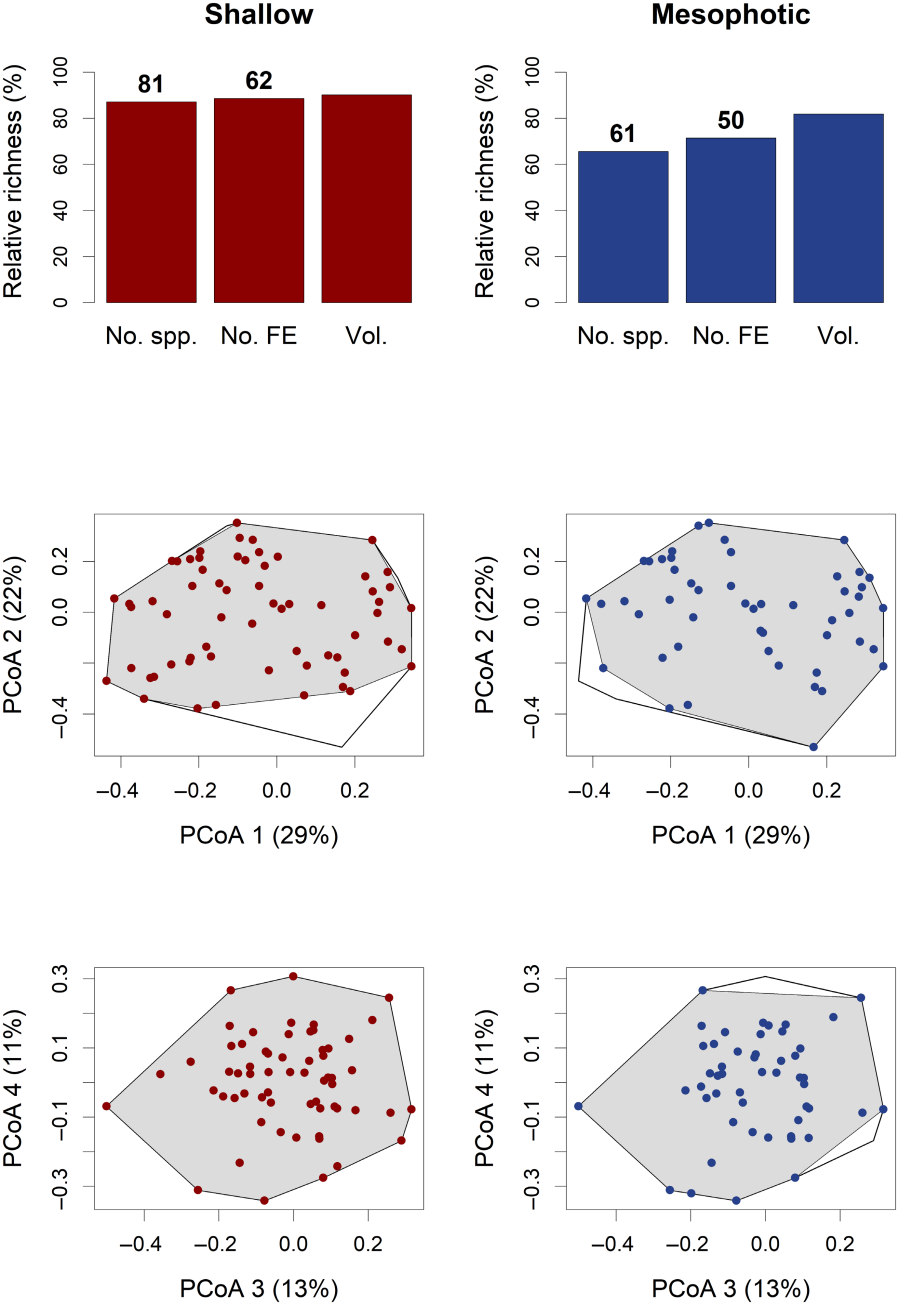
Taxonomic and functional indices of the ichthyofauna in *Parque Nacional Zona Marina del Archipiélago de Espíritu Santo* (PNZMAES) between shallow and mesophotic reefs. In the first‐row, histograms of species richness (No. spp.), number of functional entities (No. FE), and functional volume occupied percentage (Vol.) are presented (absolute values are depicted on each bar). The second and third rows show the distribution of FEs on axes 1 and 2 and axes 3 and 4 of the space constructed using the Principal Coordinate Analysis (PCoA). The total functional volume during the study period is framed in black, whereas the functional volume per zone is presented on a gray background.

**FIGURE 4:**
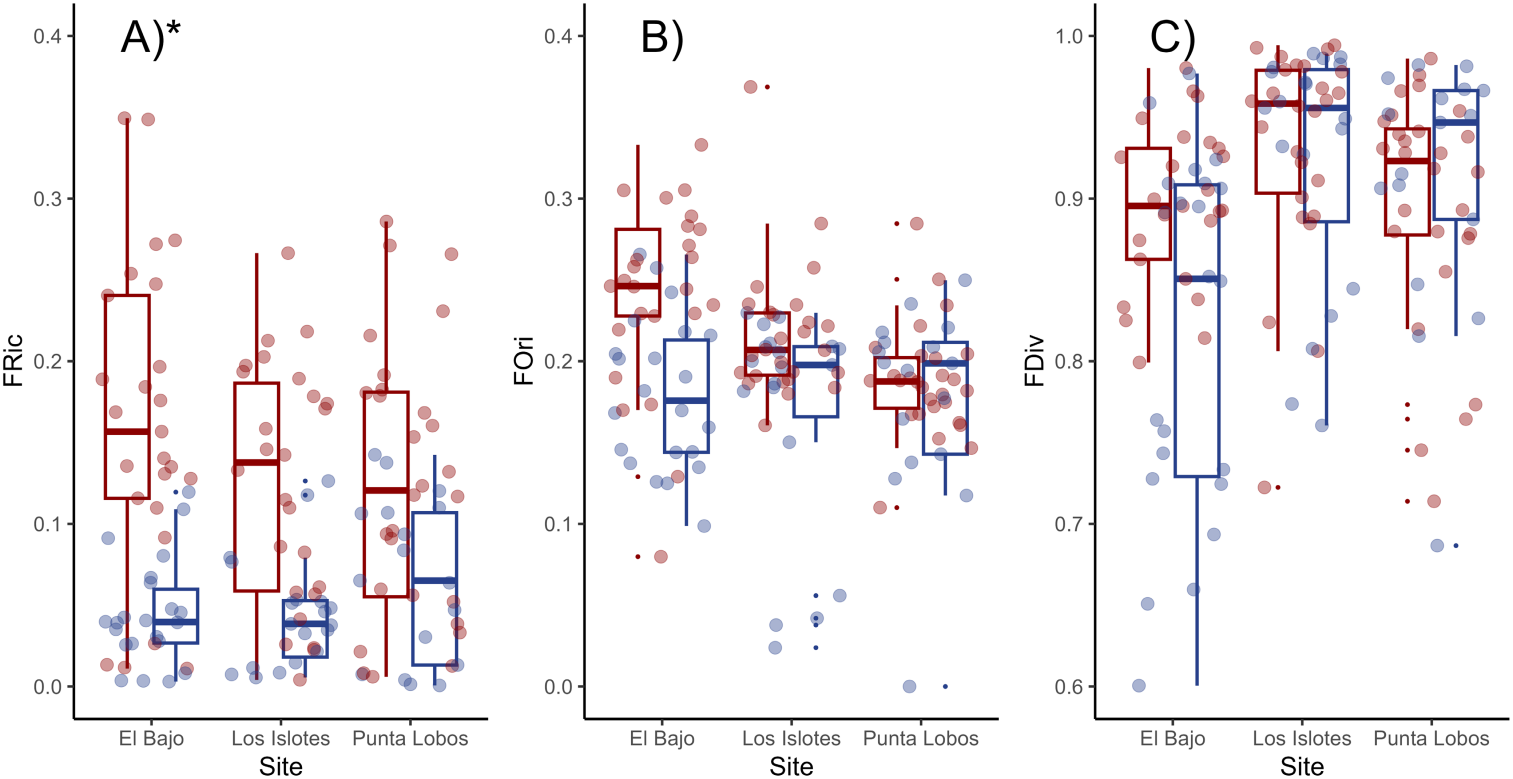
Boxplot for (A) functional richness (FRic), (B) functional originality (FOri), and (C) functional divergence (FDiv) of the ichthyofauna recorded per video transect in shallow (red) and mesophotic reefs (blue) in *Parque Nacional Zona Marina del Archipiélago de Espíritu San*to (PNZMAES) during 2021–2022. The rectangle (box) depicts the interquartile range; vertical lines (whiskers) extend to maximum and minimum values; horizontal lines within the box represent the median, and extreme solid dots are considered outliers. Asterisks highlight significant differences between sites.

With regard to the functional structure of fish assemblages per zone (Figure 3), higher values were observed in the shallow zone (No. FE = 62; Vol. = 90%) compared with those registered in the mesophotic zone (No. FE = 50; Vol = 82%). Also, FRic (mean value ± SD) was significantly higher (*p* < 0.001; Table 2) in the shallow zone (0.14 ± 0.09) than in the mesophotic zone (0.05 ± 0.04; Figure 4A). El Bajo presented the highest values for FRic at shallow depths (0.16 ± 0.10), whereas Punta Lobos presented the highest values for FRic in mesophotic reefs (0.07 ± 0.05), when compared with the remaining sites (< 0.05). FOri and FDiv (Figure 4B,C) showed no differences between zones (*p* > 0.05; Table 2), which indicates that both depth strata exhibited similar redundancy and abundance distributions among the FEs present.

**TABLE 2:** Generalized Linear Mixed Models results accounting for differences in fish diversity between shallow and mesophotic zones in *Parque Nacional Zona Marina del Archipiélago de Espíritu Santo* (PMZMAES).

	Estimate	Standard Error	Test value	*p*
**FRic**				
Intercept	14.30	2.34	6.11	< 0.001*
Shallow zone	7.40	1.59	4.65	< 0.001
**FOri**				
Intercept	5.19	0.33	15.64	< 0.001
Shallow zone	0.74	0.46	1.58	> 0.05
**FDiv**				
Intercept	1.12	0.03	33.65	< 0.001
Shallow zone	0.02	0.013	1.95	0.05
**Taxonomic richness (*q* = 0)**				
Intercept	2.65	0.15	17.27	< 0.001
Shallow zone	−0.52	0.04	−13.61	< 0.001*
**Taxonomic entropy (*q* = 1)**				
Intercept	0.27	0.05	5.50	< 0.001
Shallow zone	0.03	0.01	2.74	< 0.001*
**Functional richness (*q* = 0)**				
Intercept	0.27	0.00	34.03	< 0.001
Shallow zone	−0.00	0.00	−0.26	> 0.05
**Functional entropy (*q* = 1)**				
Intercept	0.44	0.05	8.10	< 0.001
Shallow zone	0.00	0.01	0.34	> 0.05
Taxonomic distinctness (Δ*)				
Intercept	0.02	0.00	14.19	< 0.001
Shallow zone	0.00	0.00	0.15	> 0.05

*Note:* The generalized linear mixed models were constructed as follows: Index ~ zone + (1|year) + (1|season) + (1|site).

* Significant spatial changes according to their *p* value.

**FIGURE 5:**
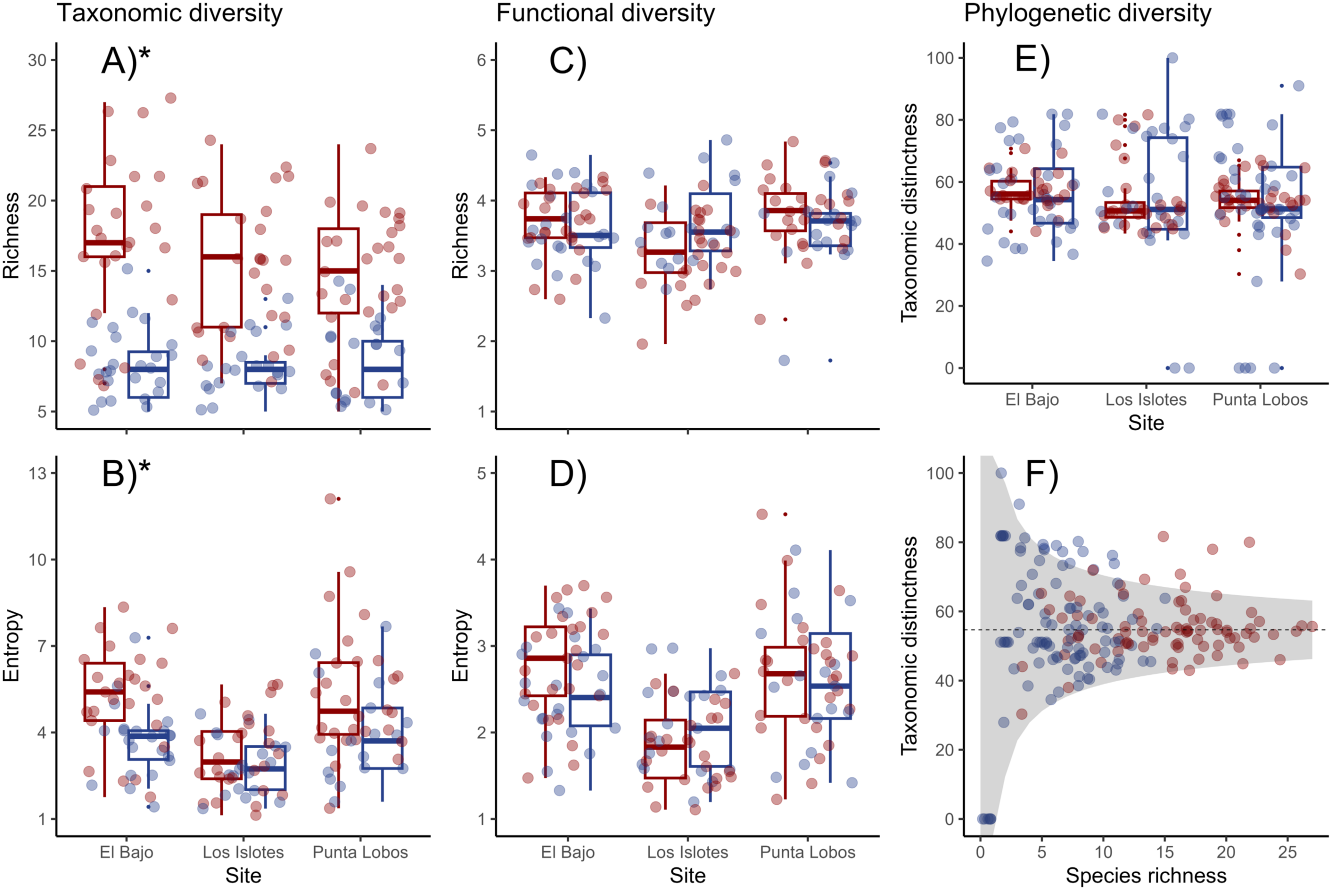
Boxplot of Hill numbers for taxonomic (A, B), functional (C, D), and phylogenetic diversity (E) of the ichthyofauna recorded per video transect in shallow (red) and mesophotic (blue) reefs in *Parque Nacional Zona Marina del Archipiélago de Espíritu Santo* (PNZMAES) during 2021–2022. (A, C) show richness; (B, D) show entropy. The rectangle (box) depicts the interquartile range; vertical lines (whiskers) extend to maximum and minimum values; horizontal lines within the box represent the median, and extreme solid dots are considered outliers. Asterisks highlight significant differences between sites. (F) Funnel plot where values of Δ* for each transect (markers) are shown. The gray area indicates the 95% confidence interval, and the dotted line constitutes the expected values for this index.

Taxonomic richness (*q* = 0; Figure 5A) and entropy (*q* = 1; Figure 5B) showed significantly higher Hill numbers (taxonomic richness [15.70 ± 5.13, *p* < 0.001]; taxonomic entropy [4.61 ± 2.05, *p* < 0.01]) in the shallow zone compared to the mesophotic zone (8.93 ± 2.25 and 3.51 ± 1.39). Nevertheless, the Hill numbers for functional diversity (Figure 5C,D) and Δ* (Figure 5E) showed similar values among shallow and mesophotic reefs, as non‐significant differences were observed (*p* > 0.05; Table 2). Moreover, the funnel plot (Figure 5F) indicated that most Δ* values were within the 95% confidence interval for both bathymetric zones. However, species in the mesophotic zone tended to be less related than expected. Altogether, these results indicated that even when the taxonomic diversity was higher in shallow depths, the mesophotic fish assemblages exhibited resemblance at functional and phylogenetic levels. These results could imply a similar range of functions and taxonomic relatedness between species in both depth strata, which could be essential to maintaining ecological reef processes in deeper habitats.

## 4 Discussion

At PNZMAES, the mean value of *K*_d490_ (> 0.07 m^−1^) and *K*_dPAR_ (> 0.13 m^−1^) was similar among study sites, which agrees with the values of Case I waters dominated by phytoplankton with low proportions of dissolved inorganic and organic matter (Lee and Tang 2022; Morel et al. 2007) and those of continental waters influenced by seasonal upwelling that reduces light availability (Pérez‐Castro et al. 2022). These seasonal changes in water transparency were reflected in the bathymetric range of the mesophotic zone, as northerly winds and the presence of GCW favor ocean mixing and elevated nutrient concentrations and primary productivity at the surface during the cold season (Herrera‐Cervantes 2019; Obeso‐Nieblas et al. 2007). Therefore, a shallower and narrower mesophotic zone was observed (11–37 m) during the cold season, whereas a more profound and more expansive mesophotic zone (21–67 m) was observed in the warm season, which was probably associated with high stratification, low nutrient concentrations, and the presence of TSW (Coria‐Monter et al. 2014; Norzagaray‐López et al. 2017). These results suggest that light penetrated throughout the water column, thus the location of the mesophotic zone in PNZMAES was highly variable and depended on water turbidity and seasonal water temperature changes (Kahng et al. 2019). Therefore, light attenuation analyses should be included in future ecological studies to determine the upper limit of the mesophotic zone (21 m in this study) with greater accuracy instead of assuming the usual depth of 30 m (Kahng et al. 2019).

The number of species registered in this study (*S* = 96 species) was similar to the richness reported in previous studies that have evaluated shallow reefs in PNZMAES with visual underwater census methods (*S* = 75 species, Pérez‐España et al. 1996; *S* = 80 species, Arreola‐Robles and Elorduy‐Garay 2002; *S* = 120 species, Rodríguez‐Romero et al. 2005). However, previous studies conducted in deeper reefs with ROVs have reported fewer species (*S* = 66 species, Hollarsmith et al. 2020; *S* = 61 species, Silva‐Montoya, Ramírez‐Ortiz, and Calderon‐Aguilera 2024; *S* = 73 species, Velasco‐Lozano et al. 2020). These differences could be associated with several factors, such as the survey methods and frequency, the survey depths and sites, and the inclusion of different species groups with distinct behaviors (e.g., elasmobranchs or cryptic fishes; Pyle et al. 2019).

The high proportion of shared species (*S* = 52 species) and the similar values of species richness between shallow (*S* = 81 species) and mesophotic (*S* = 67 species) zones in PNZMAES, as observed in the Venn diagram (Appendix S4), could be associated with environmental and habitat resemblance due to spatial proximity and the presence of rocky reefs in both strata (Aburto‐Oropeza and Balart 2001). This result does not agree with those reports in oligotrophic regions where fish species richness has been found to decrease in mesophotic depths (~30 m to 150 m), which is the result of lower coral cover due to the diminished light intensity of deeper areas (Andradi‐Brown et al. 2016; Kahng et al. 2019; Pyle et al. 2019). Thus, the fact that that we did not observe an effect of the vertical complexity (measured as the presence of soft corals, black corals, and hard corals), in addition to the report that at the Eastern Tropical Pacific and Gulf of California, reef fish diversity does not relate directly with coral cover (Aguilar‐Medrano and Calderon‐Aguilera 2016; Cortés et al. 2017; Olán‐González et al. 2020), could increase the potential of mesophotic reefs to function as refuges for certain species in the face of recent massive coral bleaching events reported within the region (López‐Pérez et al. 2023).

The number of functional entities (shallow = 62; mesophotic = 50) and their dominant biological traits (medium‐large size, high mobility, and invertivore and piscivore diets) were similar between zones. Generalist species in both strata, such as *C. colonus* and *L. argentiventris* , exhibit features that allow them to migrate and take shelter in the mesophotic zone; however, exclusive shallow species and their biological traits must be analyzed to identify particular functions that could be threatened in the face of future environmental and anthropogenic disturbances in PNZMAES (Bridge et al. 2016; Luiz et al. 2013; Myers et al. 2020). According to the results of the GLM (Table 1), reef fish composition varied statistically over space (site and zone) and time (season and year). The observed spatiotemporal dissimilarities could be related to local environmental variations and oceanographic events, such as fluctuating temperatures and internal waves, which affect fish distributions throughout the water column (Galland, Hastings, and Leichter 2019; Pinheiro et al. 2019). Hence, it is necessary to continue monitoring shallow and deep habitats to evaluate if habitat and environmental variability affect fish diversity (Chaikin, Dubiner, and Belmaker 2022; Nielsen et al. 2021).

Although more species and functional entities were recorded in the shallow zone compared to the mesophotic zone (Figure 3), the mesophotic fish assemblages covered more than 80% of the total functional volume. This result implies that most of the critical functions in shallow reefs are maintained in mesophotic habitats despite differences in species richness, as has been observed in global studies of shallow reefs that have reported similar functional volumes (> 60%) between regions of low fish diversity and biodiversity hotspots (Mouillot et al. 2014). Moreover, tropical and subtropical reef studies have suggested that the high resemblance in the number of FEs and functional volumes between zones indicates that similar functions are performed and that the mesophotic reefs could be considered functional refuges, where fish assemblages could withstand disturbances that affect shallow zones (Carrington et al. 2021; Loiseau et al. 2022). Nonetheless, the differences in fish species composition between zones suggest that deeper ecosystems act primarily as partial refugia to fish species that are shared between zones (52 species in this study) and that can migrate to these zones from shallow ecosystems (Bongaerts et al. 2010; Glynn 1996; Loiseau et al. 2022).

Overall, we found that for spatial studies, taxonomic indices are more sensitive than the functional and phylogenetic indices, which may be associated with the species groupings that are necessary to perform analyses with the functional and phylogenetic approach. Nonetheless, assessing these three components is crucial to expand our fundamental understanding of the function–biodiversity relationship and to incorporate commonly measured and rarely measured biological traits that are relevant to ecosystem processes (Bosch et al. 2021; Le Bagousse‐Pinguet et al. 2019). The fact that FRic (Figure 4A) as well as the Hill numbers for taxonomic richness and entropy (Figure 5A,B) were significantly lower in mesophotic reefs than in shallow reefs (*p* < 0.01; Table 2) suggests that deep fish assemblages are less diverse and more homogenous due to the dominance of fewer species with similar combinations of traits (i.e., the presence of generalist species). This is consistent with previous studies, which have reported that large size, high mobility, and high trophic level are traits that are favored globally and regionally (Luiz et al. 2013; Parravicini et al. 2021; Thomson and Gilligan 2002). Similar trends of decreasing biodiversity with increasing depth have been associated with lower habitat complexity (Olán‐González et al. 2020; Pinheiro et al. 2023; Hollarsmith et al. 2020). However, we did not find a significant effect of substrate and vertical complexity on fish diversity. Thus, future studies should determine how resources and habitats in mesophotic ecosystems change, particularly in El Bajo and Los Islotes, which were responsible for the largest contrasts between zones.

Conversely, FOri and FDiv (Figure 4B,C), along with the Hill numbers for functional richness and entropy (Figure 5C,D) and Δ* (Figure 5E), were not significantly different between zones (*p* > 0.05; Table 2), which indicates that the overall distribution of FEs and their abundance is similar (Appendix S3). The high values observed for FDiv (> 0.81) suggest that fish assemblages use resources efficiently and evenly throughout the water column on the eastern coast of PNZMAES. In contrast, the values for FOri (< 0.25) indicate low redundancy, meaning that a small proportion of species can substitute their functional equivalents in the case of their loss (Rosenfeld 2002; Mason et al. 2005; Mouillot et al. 2013; Francisco and de la Cueva 2017). Moreover, similar values of functional and phylogenetic diversity may indicate that species with similar traits (measured and unmeasured) are replaced in deeper reefs, possibly related to limited resource competition between shallow and mesophotic assemblages, which ultimately might allow the coexistence of evolutionarily and functionally similar species under future conditions (Bosch et al. 2021; Goetze et al. 2021). These results support our hypothesis of the existence of resemblance in fish phylogenetic and functional diversity between zones. However, taxonomic diversity was different between zones, which indicates that deep reefs could serve as partial refugia only in the face of current disturbances in shallow zones.

In conclusion, water clarity near the eastern coast of PNZMAES presented temporal variability associated with upwelling events and phytoplankton growth during the 2021–2022 period, which led to seasonal differences in the bathymetric range of the mesophotic zone. Although fish diversity between shallow and mesophotic reefs indicated a high proportion of shared species, fish assemblages appeared less diverse in deeper reefs. Decreasing diversity at increasing depth may be due to the less environmental variations and resource availability at deep strata. Nevertheless, mesophotic reef fish assemblages are evolutionarily and functionally similar to their counterparts, which suggests that they maintain the most critical functions performed in shallow reefs and could be considered partial refuges. Finally, these results highlight the need to characterize and evaluate mesophotic ecosystems in PNZMAES with different approaches (e.g., technical diving, baited remote underwater videos, and eDNA) to determine the relationships between habitat and fish diversity, the connectivity between shallow and mesophotic reefs, and the refuge potential for different species (e.g., cryptic species with theoretically lower dispersal capabilities).

## Author Contributions

**Manuel Francisco Velasco‐Lozano:** data curation (lead), formal analysis (equal), methodology (equal), visualization (lead), writing – original draft (lead), writing – review and editing (equal). **Georgina Ramírez‐Ortiz:** conceptualization (supporting), formal analysis (lead), supervision (equal), validation (equal), writing – original draft (supporting), writing – review and editing (equal). **Luis Eduardo Calderon‐Aguilera:** conceptualization (lead), formal analysis (equal), funding acquisition (lead), project administration (lead), supervision (equal), writing – review and editing (equal). **Benjamín Alonso Martínez‐Garza:** data curation (equal), methodology (equal), visualization (supporting), writing – review and editing (equal). **Omar Valencia‐Méndez:** investigation (equal), supervision (equal), writing – review and editing (equal).

## Conflicts of Interest

The authors declare no conflicts of interest.

## Supporting information


Appendices S1–S4

## Data Availability

All authors agreed to archive data and coding in a publicly available repository in GitHub (https://github.com/Kromorg/potential‐refuge‐goc).
