# Supplementary material for: Beyond the Surface: Mesophotic Reefs as Potential Refuges for Shallow Fish Assemblages
Source: Ecol Evol. 2024 Nov 28;14(12):e70619. doi: 10.1002/ece3.70619 (PMC11602671; doi:10.1002/ece3.70619)
Supplement: Supplementary file 1 — Appendices S1–S4 [file ECE3-14-e70619-s001.docx]

**Appendix**

**Beyond the Surface: Mesophotic Reefs as Potential Refuges for Shallow Fish Assemblages**

**Authors:** Manuel Francisco Velasco-Lozano^1^, Georgina Ramírez-Ortiz^2^, Luis Eduardo Calderon-Aguilera^1^, Benjamín Alonso Martínez-Garza^3^, Omar Valencia-Méndez^1^

Corresponding author: Georgina Ramírez-Ortiz, [gramirezortiz@ola.icmyl.unam.mx](mailto:gramirezortiz@ola.icmyl.unam.mx)

**Institutions**

^1^ Departamento de Ecología Marina, Centro de Investigación Científica y de Educación Superior de Ensenada, Laboratorio de Esclerocronología, Ecología y Pesquerías de la Zona Costera.

^2^ Unidad Académica Mazatlán, Instituto de Ciencias del Mar y Limnología, Universidad Nacional Autónoma de México, Laboratorio de Ecología funcional & conservación marina.

^3^ División de Ciencias de la Salud, Biológicas y Ambientales, Universidad Abierta y a Distancia de México.

**Appendix S1.** Taxonomic checklist of reef fish assemblages in the shallow and mesophotic zone based on the classification of Nelson (2016) and their updated scientific name according to Eschmeyer’s catalog (Fricke et al., 2023). FE represents the alphanumeric code based on the six traits: maximum length (0–7 as 1, 7–15 as 2, 15–30 as 3, 30–50 as 4, 50–80 as 5, > 80 cm as 6); mobility (highly attached to the site as 1, mobile with a small home range as 2, mobile with a large home range as 3, highly mobile with a very large home range as 4); period of activity (day as D, night as N); gregariousness (solitary as 1, in pairs as 2, small as 3, large schools as 4); position in the water column (benthic as 1, benthopelagic as 2, pelagic as 3); and diet (herbivore-detritivore as HD, invertivore of sessile organisms as IS, invertivore of mobile organisms as IM, planktivore as Pk, piscivore as FC, omnivore as OM). Presence (1) is indicated for each zone.

|  | Family | Species | FE | Shallow zone | Mesophotic zone |
| --- | --- | --- | --- | --- | --- |
| Class Chondrichtyes  Infraclass Elasmobranchii  Order Heterodontiformes | Heterodontidae | *Heterodontus francisci* | 62N11IM |  | 1 |
| Order Squatiniformes | Squatinidae | *Squatina californica* | 61N11FC | 1 | 1 |
| Order Pristiformes | Rhinobatidae | *Zapteryx exasperata* | 61N11IM |  | 1 |
| Order Myliobatiformes | Dasyatidae | *Hypanus dipterurus* | 62D11IM |  | 1 |
|  | Urotrygonidae | *Urobatis concentricus* | 42N11IM | 1 | 1 |
|  |  | *Urobatis halleri* | 52N11IM |  | 1 |
|  |  | *Urobatis maculatus* | 42N11IM |  | 1 |
|  |  | *Urobatis* spp. | 42N11IM | 1 | 1 |
| Class Osteichthyes  Infraclass Holostei  Order Angulliformes | Congridae | *Heteroconger* spp. | 61N11FC |  | 1 |
|  | Muraenidae | *Gymnothorax castaneu* | 51D31Pk | 1 | 1 |
| Order Clupeiformes | Clupeidae | *Sardinops sagax* | 44D43Pk | 1 |  |
| Order Gonorynchiformes | Chanidae | *Chanos chanos* | 64D42OM | 1 |  |
| Order Aulopiformes | Synodontidae | *Synodus lacertinus* | 31N11FC | 1 | 1 |
| Order Holocentriformes | Holocentridae | *Myripristis leiognathus* | 32N32IM | 1 | 1 |
| Order Kurtiformes | Apogonidae | *Apogon* spp. | 21N32Pk | 1 | 1 |
| Order Gobiiformes | Pomacentridae | *Abudefduf troschelii* | 32D42Pk | 1 |  |
|  |  | *Azurina atrilobata* | 22D42Pk | 1 | 1 |
|  |  | *Chromis alta* | 22D12Pk | 1 | 1 |
|  |  | *Chromis limbaughi* | 21D31Pk | 1 | 1 |
|  |  | *Microspathodon dorsalis* | 41D11HD | 1 |  |
|  |  | *Stegastes* spp. | 21D31OM | 1 |  |
|  |  | *Stegastes flavilatus* | 21D31OM | 1 |  |
|  |  | *Stegastes rectifraenum* | 21D31OM | 1 | 1 |
| Order Blenniiformes | Blenniidae | *Ophioblennius steindachneri* | 31D11OM | 1 |  |
|  |  | *Plagiotremus azaleus* | 21D11FC | 1 |  |
| Order Carangiformes | Carangidae | *Caranx caballus* | 54D42FC | 1 | 1 |
|  |  | *Seriola lalandi* | 64D42FC |  | 1 |
|  |  | *Seriola rivoliana* | 64D32FC | 1 | 1 |
| Order Ostiophoriformes | Sphyraenidae | *Sphyraena argentea* | 64N33FC |  | 1 |
| Order Sygnathiformes | Fistulariidae | *Fistularia commersonii* | 64D12FC | 1 | 1 |
| Order Labriformes | Labridae | *Bodianus diplotaenia* | 53D11IM | 1 | 1 |
|  |  | *Bodianus pulcher* | 63D11IM |  | 1 |
|  |  | *Halichoeres dispilus* | 32D11IM | 1 | 1 |
|  |  | *Halichoeres melanotis* | 22D11IM | 1 |  |
|  |  | *Halichoeres* spp. | 32D11IM | 1 | 1 |
|  |  | *Halichoeres nicholsi* | 42D11IM | 1 |  |
|  |  | *Thalassoma lucasanum* | 22D31IM | 1 | 1 |
|  |  | *Xyrichtys* spp. | 22D11IM |  | 1 |
|  | Scaridae | *Scarus ghobban* | 63D31HD | 1 | 1 |
|  |  | *Scarus perrico* | 53D31HD | 1 |  |
|  |  | *Scarus rubroviolaceus* | 53D31HD | 1 |  |
|  |  | *Scarus* spp. | 53D33HD | 1 |  |
| Order Perciformes | Chaetodontidae | *Chaetodon humeralis* | 32D33OM | 1 | 1 |
|  |  | *Johnrandallia nigrirostris* | 32D32OM | 1 | 1 |
|  |  | *Prognathodes falcifer* | 22D12IS | 1 | 1 |
|  | Cirrhitidae | *Cirrhitichthys oxycephalus* | 21D31FC | 1 | 1 |
|  |  | *Oxycirrhites typus* | 21D11Pk |  | 1 |
|  | Haemulidae | *Anisotremus interruptus* | 53N12IM | 1 | 1 |
|  |  | *Haemulon flaviguttatum* | 43N31FC | 1 |  |
|  |  | *Haemulon maculicauda* | 33N41FC | 1 |  |
|  |  | *Haemulon sexfasciatum* | 53N31FC | 1 | 1 |
|  | Kyphosidae | *Kyphosus elegans* | 53D42HD | 1 |  |
|  |  | *Kyphosus* spp. | 53D32HD | 1 |  |
|  |  | *Kyphosus vaigiensis* | 54D32IM | 1 |  |
|  | Lutjanidae | *Hoplopagrus guentherii* | 64N11FC | 1 | 1 |
|  |  | *Lutjanus argentiventris* | 53N41FC | 1 | 1 |
|  |  | *Lutjanus guttatus* | 53N32IM | 1 | 1 |
|  |  | *Lutjanus jordani* | 54N31FC | 1 | 1 |
|  |  | *Lutjanus novemfasciatus* | 64N11FC | 1 | 1 |
|  |  | *Lutjanus* spp. | 53N31FC | 1 | 1 |
|  |  | *Lutjanus viridis* | 34N42FC | 1 |  |
|  | Malacanthidae | *Caulolatilus princeps* | 63D11IM | 1 | 1 |
|  | Mullidae | *Mulloidichthys dentatus* | 42N31IM | 1 | 1 |
|  |  | *Pseudupeneus grandisquamis* | 33N32IM |  | 1 |
|  | Pomacanthidae | *Holacanthus passer* | 42D32IS | 1 | 1 |
|  |  | *Pomacanthus zonipectus* | 42D12IS | 1 | 1 |
|  | Serranidae | *Alphestes immaculatus* | 31N11IM | 1 |  |
|  |  | *Cephalopholis colonus* | 44D42Pk | 1 | 1 |
|  |  | *Cephalopholis panamensis* | 42N11FC | 1 | 1 |
|  |  | *Epinephelus labriformis* | 53N11FC | 1 | 1 |
|  |  | *Epinephelus quinquefasciatus* | 64D11FC | 1 | 1 |
|  |  | *Liopropoma fasciatum* | 31N11IM | 1 | 1 |
|  |  | *Mycteroperca jordani* | 64D11FC | 1 | 1 |
|  |  | *Mycteroperca prionura* | 63N11FC | 1 | 1 |
|  |  | *Mycteroperca rosacea* | 63N11FC | 1 | 1 |
|  |  | *Mycteroperca* spp. | 63N11FC | 1 | 1 |
|  |  | *Paralabrax auroguttatus* | 53D11FC |  | 1 |
|  |  | *Pristigenys serrula* | 42N12FC |  | 1 |
|  |  | *Pronotogrammus multifasciatus* | 31D12Pk | 1 | 1 |
|  |  | *Rypticus* spp. | 31N11FC | 1 |  |
|  |  | *Rypticus bicolor* | 31N11FC | 1 | 1 |
|  |  | *Serranus psittacinus* | 33N11FC | 1 | 1 |
| Order Scorpaeniformes | Scorpaenidae | *Scorpaena guttata* | 41N11FC |  | 1 |
|  |  | *Scorpaena* spp. | 41N11FC | 1 | 1 |
| Order Acanthuriformes | Acanthuridae | *Acanthurus xanthopterus* | 53D31HD | 1 | 1 |
|  |  | *Prionurus laticlavius* | 53D41HD | 1 | 1 |
|  | Zanclidae | *Zanclus cornutus* | 32D31IS | 1 | 1 |
| Order Tetraodontiformes | Balistidae | *Balistes polylepis* | 53D11IM | 1 | 1 |
|  |  | *Pseudobalistes naufragium* | 63D12IM | 1 |  |
|  |  | *Sufflamen verres* | 43D31IM | 1 | 1 |
|  | Diodontidae | *Chilomycterus reticulatus* | 52N11IM | 1 |  |
|  |  | *Diodon holocanthus* | 42N11IM | 1 | 1 |
|  |  | *Diodon hystrix* | 42N11IM | 1 |  |
|  | Ostraciidae | *Ostracion meleagris* | 32D11OM | 1 |  |
|  | Tetraodontidae | *Arothron meleagris* | 42D12IS | 1 | 1 |
|  |  | *Canthigaster punctatissima* | 21D11IS | 1 | 1 |

**Appendix S2.** Upper and lower limit estimates of the mesophotic zone at PNZMAES, based on light attenuation of photosynthetically active radiation (K_dPAR_) recorded during 2021 and 2022 through remote sensors. Rows in black show the months with higher and lower light absorption in the study region

| **Month** | **K_dPAR_ (m^-1^) range** | **Upper limit z_10%_-z_1%_ (m)** | **Lower limit z_1%_ + 1-z_0.1%_ (m)** |
| --- | --- | --- | --- |
| Los Islotes |  |  |  |
| **January** | **0.16-0.16** | **14-28** | **29-42** |
| **February** | **0.17-0.21** | **11-24** | **25-40** |
| **March** | **0.18-0.18** | **12-25** | **26-38** |
| April | 0.17-0.17 | 13-27 | 28-41 |
| May | 0.15-0.15 | 15-30 | 31-45 |
| June | 0.13-0.18 | 12-29 | 30-51 |
| July | 0.12-0.13 | 18-37 | 38-58 |
| **August** | **0.10-0.10** | **22-44** | **45-67** |
| **September** | **0.10-0.10** | **22-45** | **46-69** |
| **October** | **0.09-0.09** | **24-48** | **49-73** |
| November | 0.10-0.12 | 19-41 | 42-67 |
| December | 0.14-0.14 | 16-32 | 33-48 |
|  |  |  |  |
| Punta Lobos |  |  |  |
| **January** | **0.17-0.19** | **12-26** | **27-40** |
| **February** | **0.17-0.20** | **11-25** | **26-40** |
| **March** | **0.18-0.18** | **13-25** | **26-38** |
| April | 0.17-0.17 | 13-27 | 28-41 |
| May | 0.15-0.15 | 15-31 | 32-47 |
| June | 0.12-0.17 | 13-31 | 32-58 |
| July | 0.11-0.13 | 17-38 | 39-61 |
| **August** | **0.10-0.10** | **22-45** | **46-68** |
| **September** | **0.10-0.11** | **21-43** | **44-68** |
| **October** | **0.09-0.09** | **24-49** | **50-74** |
| November | 0.10-0.12 | 19-42 | 43-70 |
| December | 0.14-0.14 | 16-32 | 33-50 |
|  |  |  |  |
| El Bajo |  |  |  |
| **January** | **0.16-0.217** | **14-28** | **29-43** |
| **February** | **0.16-0.16** | **15-29** | **30-44** |
| **March** | **0.17-0.18** | **13-26** | **27-40** |
| April | 0.14-0.16 | 15-30 | 31-48 |
| May | 0.12-0.13 | 18-37 | 38-55 |
| June | 0.10-0.14 | 17-38 | 39-66 |
| July | 0.10-0.11 | 21-44 | 45-69 |
| **August** | **0.09-0.09** | **25-49** | **50-74** |
| **September** | **0.09-0.10** | **23-47** | **48-73** |
| **October** | **0.09-0.09** | **25-51** | **52-78** |
| November | 0.10-0.12 | 19-43 | 44-72 |
| December | 0.13-0.15 | 16-33 | 34-51 |


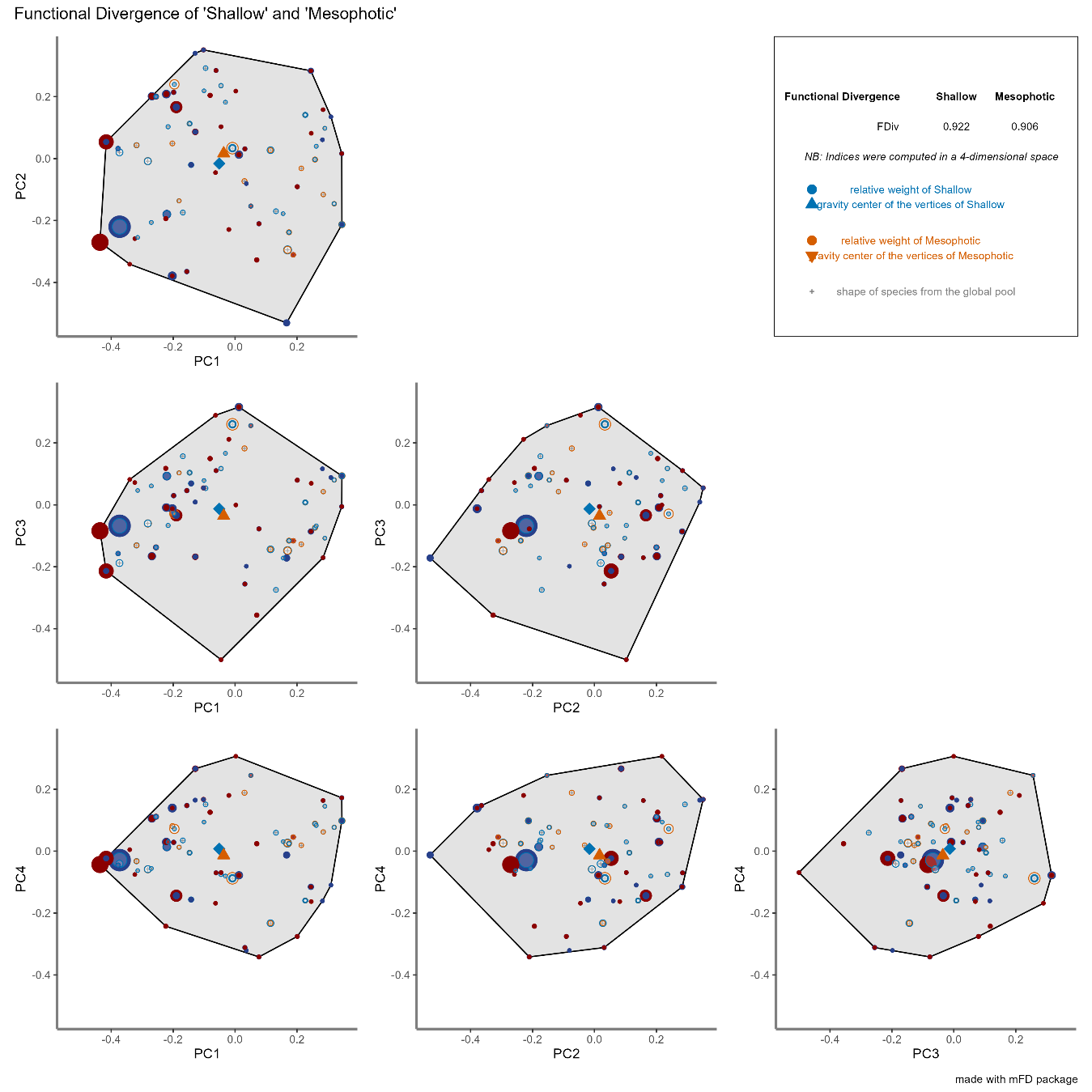


**Appendix S3.** Functional entities’ (FEs) distribution, weighted by the abundance (circle size) of the ichthyofauna at shallow (red) and mesophotic (blue) reefs at all four axes of the functional space built with the PCoA. Functional volume during the study period is depicted with a grey background, along with the gravity center for shallow (blue diamond) and mesophotic (orange triangle) zones.


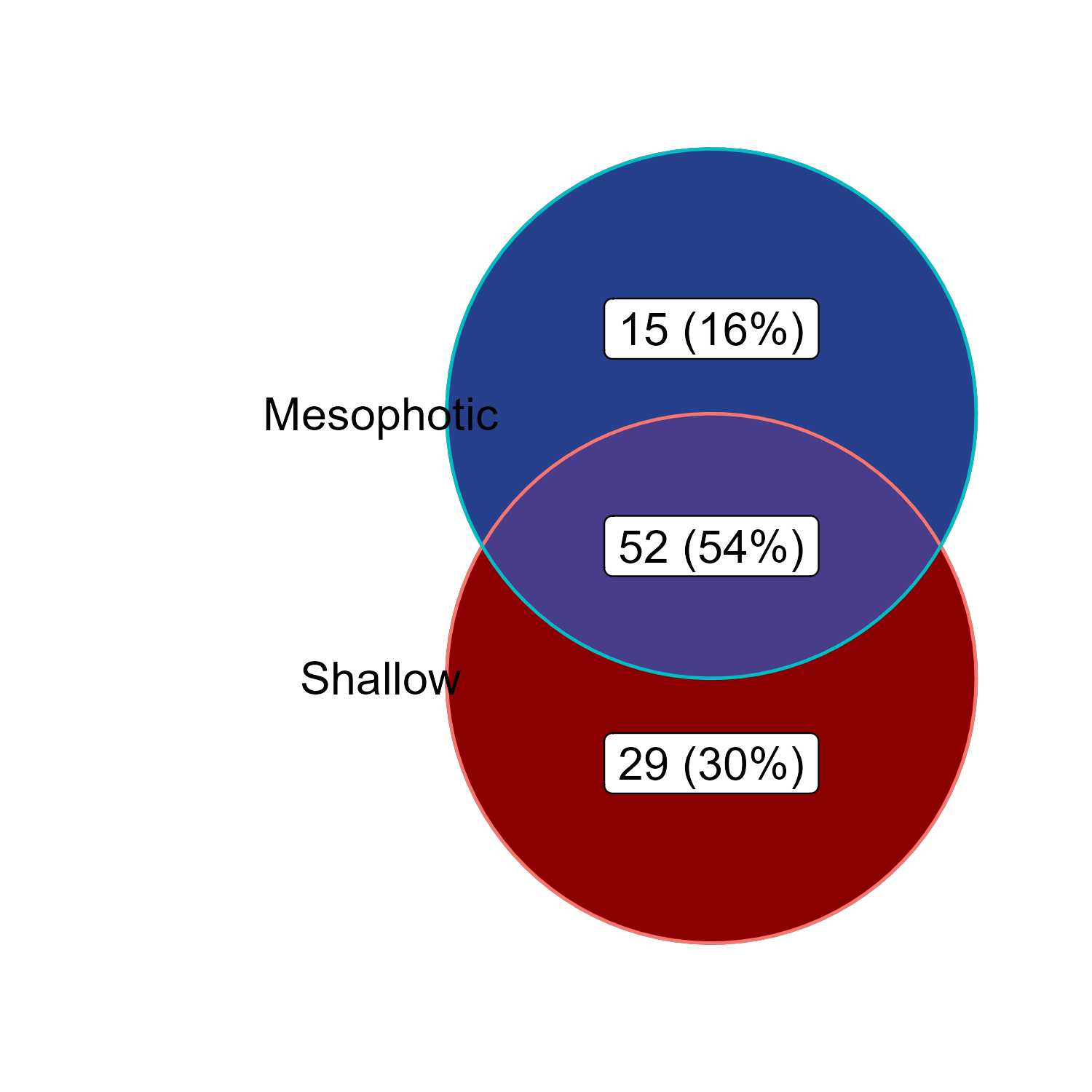


**Appendix S4.** Venn diagram depicting the number and percentage of exclusive and shared species among shallow and mesophotic zones.
